# Physical pain and risk of suicide and non-fatal self-harm among Swedish construction workers followed over three decades

**DOI:** 10.1186/s12995-026-00532-6

**Published:** 2026-10-12

**Authors:** Kirsten Mehlig, Hannah Sundvall, Ingibjörg H. Jonsdottir, Jenny Nyberg, Leo Stockfelt, Margda Waern, Maria Åberg

**Affiliations:** 1 Biostatistics, School of Public Health and Community Medicine, Institute of Medicine, Sahlgrenska Academy, University of Gothenburg, Box 469, Gothenburg, SE – 405 30 Sweden; 2 Institute of Stress Medicine, Region Västra Götaland, Gothenburg, Sweden; 3 Occupational and Environmental Medicine, School of Public Health and Community Medicine, Institute of Medicine, Sahlgrenska Academy, University of Gothenburg, Gothenburg, Sweden; 4 Occupational and Environmental Medicine, Sahlgrenska University Hospital, Gothenburg, Sweden; 5 Department of Psychiatry and Neurochemistry, Institute of Neuroscience and Physiology, Sahlgrenska Academy, University of Gothenburg, Gothenburg, Sweden; 6 Region Västra Götaland, Sahlgrenska University Hospital, Psychosis Clinic, Gothenburg, Sweden; 7 General Practice/Family Medicine, School of Public Health and Community Medicine, Institute of Medicine, Sahlgrenska Academy, University of Gothenburg, Gothenburg, Sweden; 8 Region Västra Götaland, Regionhälsan, Gothenburg, Sweden

## Abstract

**Aim:**

This observational cohort study aimed to investigate the extent to which physical pain increases the long-term risk of suicidal behavior among construction workers.

**Methods:**

In 1989-93, 79,099 men from the Swedish construction workers cohort completed a questionnaire on working conditions and physical symptoms, including pain, in addition to their routine health examination. The participants assessed how often they had experienced pain in nine anatomical areas over the past year; this was summarized into four categories describing pain in the neck, shoulders and upper back; the lower back and hip; the upper limbs (hand, wrist, elbow); and the lower limbs (foot, ankle, knee). Frequent pain in at least one location, as well as location-specific frequencies, were considered main exposures. Multivariable Cox proportional hazard regression was used to examine associations between pain and risk of (1) suicide, and (2) non-fatal self-harm including suicide attempt, censored at emigration, disability pension, retirement, other causes of death and end of follow-up in 2018.

**Results:**

55% of workers reported frequent pain in at least one location, with lower back being the most common site. Frequent pain increased the risk of non-fatal self-harm by 25% compared to no pain in any location and the risk of suicide was doubled. Frequent pain in hand, wrist or elbow was associated with excess risk of both fatal and non-fatal self-harm, while frequent pain in foot, ankle or knee was associated with non-fatal self-harm only, independent of socio-demographic factors, parental origin, body mass index, height and blood pressure. After adjustment for occupational group and unfavorable working position, the increased risk associated with pain in the limbs remained.

**Conclusion:**

Our findings underscore the importance of recognizing and addressing the psychological consequences of physical pain among construction workers, particularly when the pain affects the arms or legs.

**Supplementary Information:**

The online version contains supplementary material available at https://doi.org/10.1186/s12995-026-00532-6.

## Introduction

Numerous population-based studies have shown that suicidal behaviour is more common among construction workers than in the general working population [1–6]. This male-dominated occupation is characterized by several interrelated psychological and somatic factors that increase the risk of suicide. Accidents and heavy manual labour can lead to chronic pain and musculoskeletal disorders [7–10], which have been shown to have psychological consequences. A recent study estimated the annual economic burden of suicidal behaviour in Sweden at approximately €970 million, with suicide accounting for most of this cost, mainly through productivity losses [11]. Among Swedish construction workers, almost 20% received disability pension before the age of 65, with the highest proportion recorded for rock workers (37%) and crane operators (34%), suggesting an impact of heavy manual labour and non-ergonomic working postures on health [12]. Rock workers constituted the occupational group with the greatest number of days lost due to disability pension [12] as well as the highest risk of suicide [13].

Growing attention has been given to the comorbidity and bidirectional relationship between mental health and pain, particularly because their combined social disadvantages may entail even greater costs [14]. Chronic pain is associated with mental health disorders such as anxiety and depression [15–17], and with suicidal thoughts and suicidal behavior in the general population [18–24] independent of concomitant mental disorder [25]. Among construction workers, it has been shown that a high incidence of injuries and pain are significant risk factors for suicide among roofers [26], and severe pain resulting from workplace accidents was one of several factors identified as a major source of stress leading to suicide among construction workers in general [27]. In contrast to suicide, the link between physical pain and non-fatal self-harm has so far been little researched [28] even though non-fatal self-harm is a strong risk factor for suicide [29]. The study of physical pain and self-harming behaviour is of particular importance in the construction sector, where work continues to be perceived as hierarchical and inherently stressful, with tough communication as part of the macho culture and widespread stigma that prevents workers from seeking help for health problems of any kind [30].

The overall aim of this study was to assess the prevalence of physical pain and its association with suicidal behavior in a nationwide cohort of construction workers in Sweden. Specifically, this exploratory study aimed to investigate the prospective associations between pain in four body regions - the neck, shoulders and upper back, the lower back and hips, as well as upper and lower limbs - and two types of suicidal behavior, namely non-fatal self-harm and completed suicide, whilst taking into account the influence of potential confounding factors. By clarifying the role of site-specific pain in relation to fatal and non-fatal self-harm among construction workers, this study addresses an important knowledge gap, which is of direct relevance to prevention measures for this occupational group.

## Methods

### Study population

The Swedish Construction Workers Cohort (CWC) comprises observations of 389,132 workers who participated in free health examinations offered by the National Occupational Health Service (Bygghälsan) between 1971 and 1993, in which approximately 80% of employees in the construction sector participated at least once [12, 31, 32]. During the last five years of this initiative (1989–1993), a questionnaire on working conditions was distributed in connection with the regular examination. This questionnaire was returned by 87,105 participants, which corresponded to 56% of construction workers examined during these years. The examination during which the questionnaire was completed will be referred to as the baseline examination of this observational cohort study. All participants were informed that the data might be used for research purposes and gave their verbal consent. To comply with the increasingly stringent requirements regarding informed consent, the steering group (which also includes trade union representatives) approved all the studies carried out. Further, notifications in the trade press clarified that CWC participants were free to withdraw their data from the register, but no one has done so to date.

### Baseline data

Information on age as well as year and region where the baseline examination was performed was available for all participants. More than 200 job titles combined into 22 occupational groups [12, 13, 31] were further concatenated to 17 groups to account for the smaller sample size in the present study. As physical pain may be exacerbated by excess body weight, which has been associated with suicidal behavior in some studies [33–35], we adjusted for body mass index (BMI) given by measured values for weight over height squared. BMI was categorized into underweight (BMI < 18 kg/m^2^), normal weight (18 – <25 kg/m^2^), overweight (25 – <30 kg/m^2^), and obesity (≥ 30 kg/m^2^). Low resting systolic blood pressure (SBP) has been shown to be associated with higher pain sensitivity [36] and with poorer mental health outcomes including depression and anxiety disorders [37]. Therefore, SBP was included as a potential confounder of the association between pain and suicidal behavior. Height was included as a correlate of blood pressure, and due to an inverse association with suicide risk [38]. Information on parental origin and the participant’s education and marital status was obtained from the Longitudinal Integrated Database for Health Insurance and Labour Market Studies (LISA), which is updated annually by Statistics Sweden (SCB). Parental origin was parametrized as the number of parents born in Sweden (none, 1, 2). Education may account for differences in cognitive reserve and socioeconomic status and was categorized into low (mandatory education), medium (high school), and high (university). Marital status was included as a measure for social integration [39] and distinguished between unmarried, married, divorced and widowed. Due to low frequency (0.4% of the total population), ‘widowed’ was combined with ‘married’ in the subsequent analyses. Smoking status was recorded as ‘current smoker’ or ‘non-smoker’; however, as it might be a consequence of pain rather than a risk factor for it, it was not included in the group of potential confounding factors such as anthropometric data, parental origin, educational attainment and marital status. Instead, current smoking was used here to assess the internal validity of the question regarding physical pain. This also applies to diagnoses of alcohol-use disorder (AUD) prior to the baseline examination, which were taken from the national hospital register (ICD-10: F10; ICD-9: 291, 303, 305; ICD-8: 291, 303).

### Questionnaire data

**Fig. 1:**
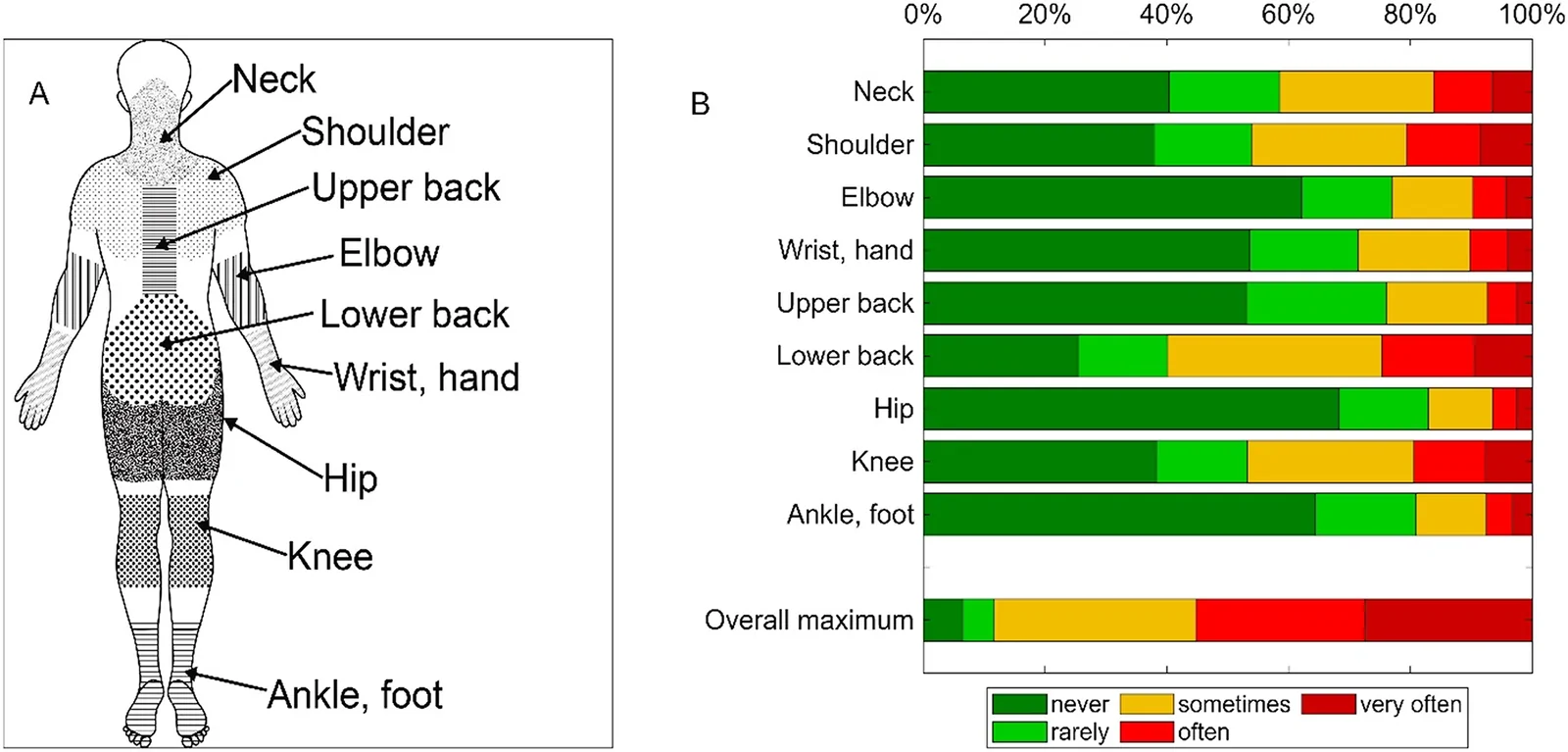
The pain locations indicated in the questionnaire (**A**) and the distribution of pain frequency per location as well as the overall maximum (**B**)

Five categories of pain frequency over the past year (never, rarely, sometimes, often, very often) were reported for nine anatomical locations (Fig. 1A). One exposure variable was the highest level of pain frequency in at least one location, hereafter referred to as ‘maximum pain frequency in at least one location’, and categorized as frequent (often, very often), occasional (sometimes, rarely) and no experience of pain. To examine site-specific pain whilst controlling for the risk of a type 1 error, the nine pain locations were grouped into four regions, (1) neck, shoulder and upper back, (2) elbow, hand/wrist, (3) lower back and hip, and (4) knee and foot/ankle. The highest level of pain frequency in each region was categorized as frequent, occasional and no experience of pain.

The questionnaire also comprised four questions on the current frequency of unfavorable working postures, i.e. heavy lifting, bending forward or twisting, with hands above shoulders, in a kneeling position, with five categories dichotomized into frequent versus less frequent. Finally, questions relating to sleep problems and depressive symptoms were included, distinguishing frequent versus infrequent prevalence. As they are regarded more as consequences than causes of pain, they were not included in the list of potential confounders of the risk of suicidal behaviour associated with pain; instead, they serve here to validate and characterize in greater detail the status of frequent physical pain, alongside AUD and smoking.

### Endpoint and censoring information

Data from the CWC were linked to the national hospital and cause-of-death registers using the personal identification number issued to every Swedish citizen. Diagnostic codes were selected according to the *International Classification of Diseases* (ICD-8 to ICD-10), and first diagnoses after baseline were recorded, regardless of whether they were main or contributing causes. Codes for non-fatal self-harm and suicide, both with and without established intent of self-harm, are listed in Table 1. Since some cases registered as events with undetermined intent of self-harm may have been accidental, we carried out a separate set of analyses after exclusion of the undetermined cases. Episodes of non-fatal self-harm before baseline were recorded separately and summarized in a binary indicator (yes versus no). As this study focused on suicidal behaviour among working individuals, observations were censored at age 65, the usual retirement age, or upon being granted a disability pension. Censoring was also applied at first emigration after baseline, as prospective events might otherwise be missed. Information on disability pension and emigration was obtained from the LISA database.

**Table 1:** Characteristics of outcomes in a cohort of male construction workers attending routine health examinations and followed over up to 30 years (*n* = 79,099)

Outcome ^a^	Events	Age at diagnosis	Incidence rate per 100 000 person years
		Mean (SD)	IR (95% CI)
Non-fatal self-harm	1462	46.8 (9.6)	101.1 (96.1, 106.5)
with known intent	441	45.1 (9.7)	30.3 (27.6, 33.3)
Suicide	271	48.4 (9.6)	18.6 (16.5, 20.9)
with known intent	234	48.5 (9.5)	16.0 (14.1, 18.2)

^a^ Suicidal behavior with established intent: X60–X84, Y870 (ICD-10); E95 (ICD-8/9)

Suicidal behavior without established intent: Y10-Y34, Y872 (ICD-10); E98 (ICD8/9)

### Definition of analytical sample and handling of missing data

As the study focuses on suicidal behaviour in men, we excluded observations of women (< 4% of all participants with questionnaire data). Missing answers to any of the nine questions about pain localization were imputed as ‘never occurred’ if at least one question was answered. Observations without valid answers to any of the nine questions on pain localization were excluded, as self-rated pain was the main exposure in this study. Sensitivity analyses were performed based on subjects with complete information on all nine pain questions. Missing data for unfavorable working positions, sleep problems, or depressive symptoms were imputed as ‘never occurred’ if at least one question from the respective set of questions was answered. For all other data, we applied listwise deletion of observations with missing data. Because impending mental illness may influence the self-report of pain, we have excluded all events (self-harm or censoring) before or within two years after baseline examination. As men with events within this time span were generally older, this exclusion criterion resulted in an upper age limit of 62 years for the final analysis group (*n* = 79,099, Table S1).

### Statistical methods

Baseline characteristics were reported as mean values with standard deviation (SD) for scale variables and percentages for categorical variables. Differences by categories of maximum pain frequency in at least one location were tested using the Kruskal-Wallis and the chi-square test, respectively. Multiple multinomial logistic regression was performed for maximum pain frequency in at least one location (ref = never) to estimate associations with age, SBP, height, and BMI, adjusted for calendar year and region. Results were presented as odds ratio (OR) with 95% confidence interval (CI). Further tests confirmed that there was no multicollinearity between the independent variables in relation to the five pain categories. For prospective outcomes, incidence rates with 95% CI were obtained using Poisson regression. Cox proportional hazard models were used to identify risk factors for each outcome. Survival time from baseline to the event of interest or censoring due to emigration, granting of disability pension, retirement at age 65, death due to other causes or end of follow-up (2018) was calculated. Firth bias correction was applied to account for the low number of events for the outcomes studied [40]. The proportional-hazard assumption was confirmed graphically and tested using weighted Schönfeld residuals. Results were given in terms of hazard ratios (HR) with 95% CIs. Linear regression models were carried out for outcome-specific survival time to ensure that there was no collinearity between the independent variables (variance inflation factors < 2). To account for temporal and regional differences in examination procedure or working conditions, all models were adjusted for calendar year and region of examination (model set 1). Further adjustment included parental origin, age, height, BMI, SBP, education, and marital status (model set 2), as well as unfavorable working position and occupational group (model set 3). The risk associated with specific occupational groups was compared with the overall mean to present the entire spectrum of risk estimates without singling out a particular reference occupation. All analyses were performed using SAS version 9.4 and MATLAB version R2025b. Given the exploratory nature of the association analyses, the significance level was set at 0.05 (2-sided tests), except for the comparisons between the 17 occupational groups, for which the significance level was lowered to 0.05/17 = 0.003 to account for multiple testing.

## Results

### Prevalence of frequent pain

About 55% of men in this sample of construction workers aged 16–62 (median age: 37 years) reported frequent pain in at least one location (Fig. 1B). The lower back was the most commonly affected area, followed by the neck, shoulders and knees. The prevalence estimates changed very little when observations with missing data on specific pain sites were excluded from the analyses (Table S2).

### Cross-sectional associations with pain

**Fig. 2:**
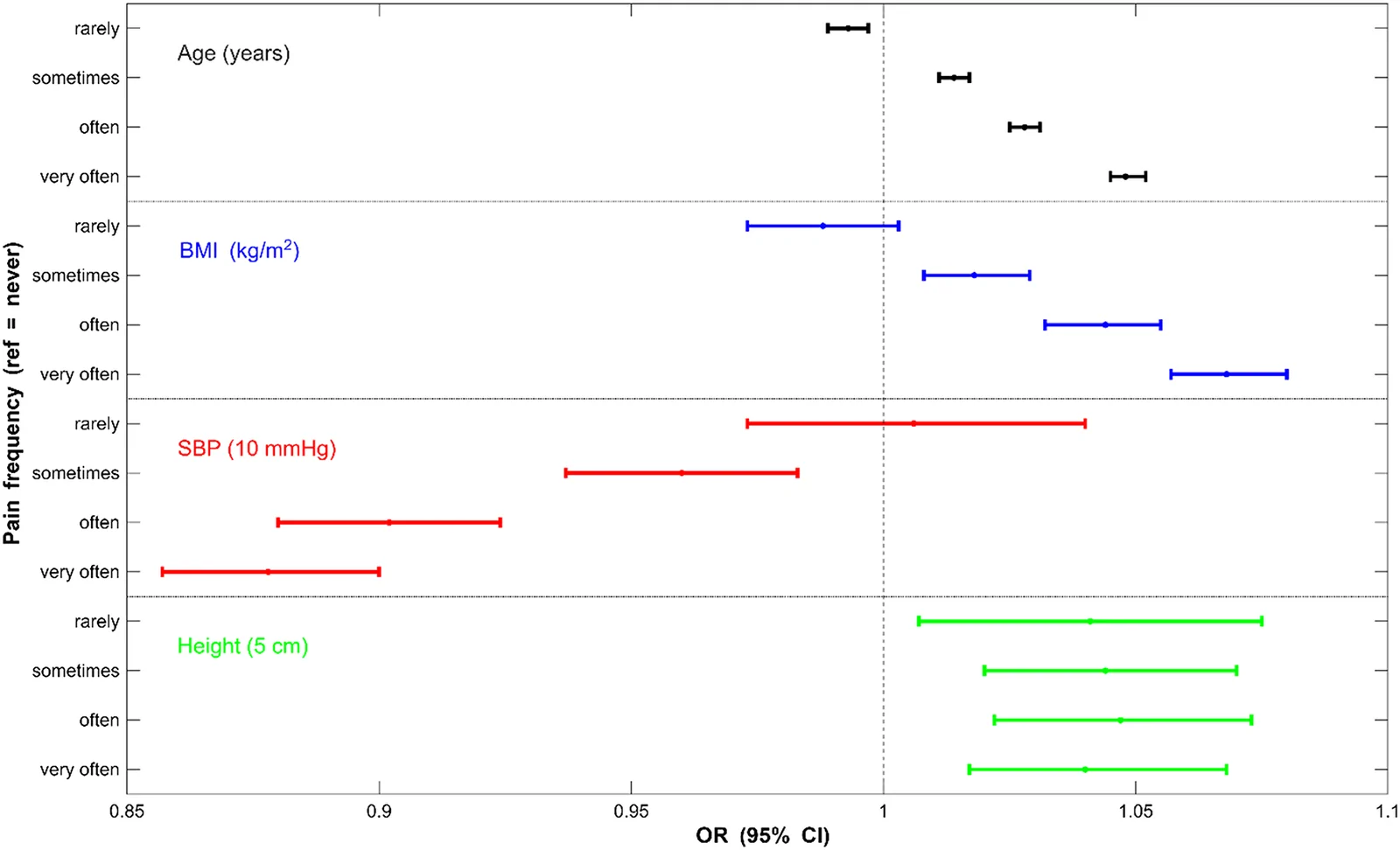
Odds ratios for maximum pain frequency in at least one location compared to no pain experience from a multivariable multinomial logistic model adjusted for age, BMI, SBP and height as well as calendar year and region of examination

Higher age and BMI were associated with a greater level of pain frequency (Table 2). Positive associations with age and BMI were also seen in a multinomial logistic regression further adjusted for SBP, height, year, and region of examination (Fig. 2). In this model, SBP was negatively associated with pain frequency, with larger effect size with respect to more frequent pain versus no pain. In contrast, greater height was associated with increased odds of any pain compared to no pain, independent of absolute pain frequency. Married or divorced men were more likely to report pain than unmarried men (Table 2). Lower education, unfavorable working positions, smoking, AUD, sleep problems, and depressive symptoms were positively associated with pain frequency.

**Table 2:** Baseline characteristics by maximum pain frequency in at least one location (*n* = 79,099)

Potential confounders for pain-related risk		Maximum pain frequency in at least one location				
	Missing observations (*n*)	Never (*n* = 5,130)	Rarely (*n* = 4,083)	Sometimes (*n* = 26,196)	Often (*n* = 21,970)	Very often (*n* = 21,720)
		**Mean (SD)**				
Age (yrs)	0	34 (12)	33 (12)	36 (12)	37 (11)	40 (11)
Height (cm)	191	178.8 (6.4)	179.2 (6.4)	178.9 (6.5)	178.7 (6.4)	178.4 (6.4)
BMI (kg/m^2^)	2,408	24.4 (3.2)	24.2 (3.0)	24.7 (3.1)	25.0 (3.1)	25.4 (3.2)
SBP (mmHg)	2,079	129.9 (13.6)	129.6 (13.6)	129.9 (13.4)	129.5 (13.5)	130.0 (13.8)
Number of parents born in Sweden	0	1.77 (0.58)	1.78 (0.56)	1.75 (0.59)	1.71 (0.63)	1.66 (0.68)
		**Column percent**				
Education:						
Low	168	25.0	23.0	29.0	32.0	38.0
High school		63.3	66.8	65.2	64.2	59.0
University		11.7	10.2	5.8	3.8	3.0
Marital status:						
Married	31	41.1	39.0	46.3	51.6	59.0
Unmarried		54.7	57.6	49.0	42.6	33.9
Divorced		4.2	3.5	4.7	5.8	7.0
Unfavorable working position (frequent vs. less often):						
Heavy lifting	869	24.0	26.5	37.2	49.7	57.7
Bending forward /twisting	869	28.8	33.2	45.3	59.4	68.3
With hands above shoulders	869	32.2	36.8	44.8	54.8	61.4
In a kneeling position	869	36.3	42.7	51.0	60.7	66.6
***Correlates of physical pain***						
Ever smoking	1974	54.0	53.1	61.2	67.1	69.7
Alcohol use disorder (AUD)	0	0.5	0.3	0.6	0.9	1.3
Sleeping problems	638	1.6	1.4	2.4	5.0	10.6
Depressive symptoms	638	0.9	0.7	1.3	2.5	4.8

Comparisons across categories of pain frequency (Kruskal-Wallis test for scale variables, chi-square test for categorical variables) were statistically significant for all variables

Body mass index (BMI), systolic blood pressure (SBP)

Among occupational groups, concrete workers, wood workers/roofers, bricklayers, floor-layers, insulation workers/plumbers, painters, and sheet-metal workers had excess risk of frequent pain while machine operators, electricians, other manual workers, and foremen/clerks had reduced risk compared to the mean risk of frequent pain in the entire cohort (Table S3). Sheet-metal workers reported frequent pain in six out of nine locations, while foremen and clerks were least likely to report frequent pain in any location. Sheet-metal workers were also among those who experienced all types of unfavorable working positions as did concrete workers, bricklayers, floor-layers, and insulation workers/plumbers (Table S4).

### Prospective associations between pain and non-fatal self-harm/suicide

The average follow-up period was 21.4 years (range 3.0–30.3 years), with the corresponding figures for cases of suicide and non-fatal self-harm being 13.8 (3.0–28.3) and 15.5 (3.0–29.1) years, respectively. The incidence of non-fatal self-harm was 101/100,000 person years, and the corresponding figure for suicide was 19/100,000. While certain (“determined”) intent of self-harm was registered in only one-third of the non-fatal cases, intent was established for 86% of all suicide deaths (Table 1).

Risk of self-harm increased across categories of pain in at least one location, with larger hazard ratios for outcomes with determined intent (Table 3, model 1 A). Frequent pain increased the risk of non-fatal self-harm by 25% and doubled the risk of suicide, compared to no pain. Of the four regions, frequent pain in the elbow or wrist/hand, as well as in the knee or ankle/foot were independently associated with a risk of non-fatal self-harm, compared with less frequent pain (Table 3, model 1B). Only frequent pain in the upper extremities was associated with an increased risk of suicide.

**Table 3:** Pain and risk of suicidal behavior adjusted for different sets of potential confounders (HR with 95% CI)

	Non-fatal self-harm	Non-fatal self-harm with known intent	Suicide	Suicide with known intent
**Model set 1: Adjustment for year, region, and age at baseline**				
Number of events /totals	1,462 /79,099	441 /79,099	271 /79,099	234 /79,099
***1 A. Maximum pain frequency in at least one location (ref = never)***				
Occasional	1.03 (0.83, 1.29)	1.17 (0.77, 1.76)	1.75 (0.93, 3.30)	1.86 (0.92, 3.76)
Frequent	**1.25 (1.01**,** 1.55)**	1.44 (0.96, 2.15)	**1.97 (1.05**,** 3.69)**	**2.13 (1.06**,** 4.27)**
***1B. Maximum pain frequency in specified regions (ref = never)***				
*Neck*,* shoulder*,* upper back*				
Occasional	0.98 (0.85, 1.12)	1.09 (0.84, 1.41)	1.33 (0.94, 1.88)	1.27 (0.88, 1.84)
Frequent	1.00 (0.85, 1.18)	1.08 (0.79, 1.46)	1.14 (0.76, 1.70)	1.11 (0.72, 1.70)
*Elbow*,* wrist/hand*				
Occasional	**1.19 (1.06**,** 1.34)**	1.11 (0.89, 1.38)	1.34 (1.00, 1.78)	1.24 (0.91, 1.69)
Frequent	**1.20 (1.02**,** 1.41)**	1.28 (0.96, 1.69)	**1.60 (1.12**,** 2.29)**	**1.57 (1.08**,** 2.30)**
*Lower back*,* hip*				
Occasional	1.03 (0.89, 1.18)	0.89 (0.69, 1.14)	0.87 (0.63, 1.21)	0.90 (0.63, 1.28)
Frequent	1.08 (0.91, 1.27)	0.93 (0.69, 1.14)	1.03 (0.71, 1.49)	1.03 (0.69, 1.54)
*Knee*,* ankle/foot*				
Occasional	1.04 (0.91, 1.18)	1.09 (0.86, 1.38)	1.03 (0.77, 1.38)	1.08 (0.78, 1.49)
Frequent	**1.22 (1.05**,** 1.41)**	**1.51 (1.16**,** 1.96)**	0.87 (0.60, 1.24)	0.99 (0.68, 1.45)
***Model set 2: Model set 1 with adjustment for parental origin***,*** SBP***,*** height***,*** BMI***,*** education***,*** marital status***				
Number of events /totals	1,430 /76,440	436 /76,440	266 /76,440	230 /76,440
***2 A. Maximum pain frequency in at least one location (ref = never)***				
Occasional	1.01 (0.81, 1.25)	1.15 (0.76, 1.73)	1.74 (0.92, 3.28)	1.85 (0.91, 3.75)
Frequent	1.23 (0.99, 1.52)	1.40 (0.93, 2.10)	**2.04 (1.08**,** 3.82)**	**2.19 (1.09**,** 4.40)**
***2B. Maximum pain frequency in specified regions (ref = never)***				
Neck, shoulder, upper back				
Occasional	0.98 (0.85, 1.13)	1.08 (0.83, 1.40)	1.27 (0.90, 1.80)	1.22 (0.84, 1.78)
Frequent	0.99 (0.84, 1.17)	1.07 (0.78, 1.45)	1.10 (0.73, 1.65)	1.07 (0.69, 1.65)
Elbow, wrist/hand				
Occasional	**1.19 (1.05**,** 1.34)**	1.09 (0.87, 1.37)	**1.36 (1.01**,** 1.82)**	1.24 (0.91, 1.70)
Frequent	**1.19 (1.01**,** 1.40)**	1.27 (0.96, 1.70)	**1.63 (1.14**,** 2.35)**	**1.58 (1.08**,** 2.32)**
Lower back, hip				
Occasional	1.02 (0.88, 1.17)	0.90 (0.70, 1.15)	0.88 (0.64, 1.23)	0.93 (0.65, 1.33)
Frequent	1.06 (0.89, 1.25)	0.92 (0.68, 1.24)	1.07 (0.73, 1.57)	1.09 (0.72, 1.64)
Knee, ankle/foot				
Occasional	1.04 (0.91, 1.18)	1.09 (0.86, 1.39)	1.05 (0.78, 1.41)	1.08 (0.78, 1.50)
Frequent	**1.21 (1.05**,** 1.41)**	**1.49 (1.14**,** 1.95)**	0.89 (0.62, 1.28)	1.01 (0.69, 1.49)
***Model set 3: Model set 2 with adjustment for occupational group and unfavorable working positions***				
Number of events /totals	1,406 /74,805	433 /74,805	260 /74,805	224 /74,805
***3 A. Maximum pain frequency in at least one location (ref = never)***				
Occasional	1.02 (0.82, 1.27)	1.10 (0.73, 1.67)	1.67 (0.88, 3.16)	1.78 (0.87, 3.62)
Frequent	1.21 (0.97, 1.51)	1.25 (0.83, 1.89)	1.87 (0.98, 3.54)	2.00 (0.98, 4.08)
***3B. Maximum pain frequency in specified regions (ref = never)***				
Neck, shoulder, upper back				
Occasional	0.98 (0.85, 1.14)	1.09 (0.84, 1.42)	1.36 (0.95, 1.94)	1.32 (0.90, 1.93)
Frequent	0.98 (0.82, 1.16)	1.04 (0.76, 1.42)	1.17 (0.77, 1.78)	1.16 (0.74, 1.81)
Elbow, wrist/hand				
Occasional	**1.19 (1.05**,** 1.34)**	1.06 (0.85, 1.33)	1.32 (0.98, 1.77)	1.20 (0.87, 1.66)
Frequent	1.17 (0.99, 1.39)	1.21 (0.91, 1.62)	**1.55 (1.07**,** 2.25)**	**1.50 (1.01**,** 2.22)**
Lower back, hip				
Occasional	1.00 (0.87, 1.15)	0.86 (0.67, 1.04)	0.85 (0.61, 1.19)	0.90 (0.62, 1.29)
Frequent	1.03 (0.87, 1.23)	0.85 (0.63, 1.15)	0.99 (0.67, 1.46)	1.01 (0.66, 1.53)
Knee, ankle/foot				
Occasional	1.05 (0.92, 1.19)	1.08 (0.85, 1.38)	1.05 (0.78, 1.43)	1.08 (0.78, 1.50)
Frequent	**1.22 (1.05**,** 1.42)**	**1.46 (1.12**,** 1.92)**	0.89 (0.61, 1.29)	1.00 (0.67, 1.48)

Cox proportional hazard regression models adjusted for different sets of baseline covariates; statistically significant results are marked in bold (*p* < .05)

Associations with maximum pain frequency category in at least one location were hardly changed by adjusting for sociodemographic and clinical confounders (Table 3, models 2 A + B). Additional adjustment for occupation and unfavorable working conditions reduced the associations with maximum pain frequency in any location, but most associations with location-specific pain frequencies remained (Table 3, model 3 A + B). Among occupational groups, road construction workers displayed increased risk for non-fatal self-harm and crane operators were at elevated risk of suicide, while electricians and white-collar workers were at reduced risk for non-fatal self-harm compared to overall risk, but the positive associations were not statistically significant after adjustment for multiple testing (not shown). Frequent heavy lifting was the only unfavorable working position, associated with an increased risk of non-fatal self-harm and suicide (not shown). However, after adjustment for occupation, the association remained for suicide only, with hazard ratios of 1.46 (1.07, 1.99) for suicide (with or without a diagnosis of determined intent) and 1.54 (1.10, 2.15) for suicide with determined intent.

Sensitivity analyses showed that the associations between lower-limb pain and non-fatal self-harm, and between upper-limb pain and suicide became stronger when participants with incomplete answers on any of the nine questions pertaining to pain locations were excluded (Table S5).

## Discussion

### Summary of main results

55% of construction workers reported frequent pain in at least one of nine anatomical locations. Frequent pain in at least one location was associated with higher risk of both non-fatal self-harm and suicide. Frequent pain was most prevalent in the lower back, but pain in other locations (elbow, hand, wrist or knee, foot, ankle) was associated with non-fatal self-harm. Only pain in the upper extremities was associated with suicide. These associations were independent of age, parental origin, height, weight, SBP, education, and marital status at the time the pain was reported. Hazard ratios were reduced after adjustment for occupational group and unfavorable working conditions, but associations with location-specific reports of frequent pain remained.

### Prevalence of frequent pain

Pain was more prevalent in specific manual occupations; for instance, 61% of bricklayers in our cohort reported frequent pain, a number that is close to results from a recent Dutch study, where 67% of brick layers reported musculoskeletal disorders [7]. A recent meta-analysis [10] provided estimates for the 1-year prevalence of musculoskeletal disorders among construction workers that were comparable with our prevalence estimates for pain experienced occasionally or frequently during the past year, with a similar pattern across the nine anatomical locations.

### Cross-sectional associations with pain

In this study, frequent pain was more prevalent in older construction workers, which is consistent with the findings of others [41, 42]. The inverse association between systolic blood pressure and perceived pain is consistent with earlier results showing that SBP was associated with better pain tolerance [36]. Our result of 10% lower odds of frequent pain per 10 mmHg strengthens the validity of the self-assessed pain exposure, while adjustment for SBP can account for individual differences in pain sensitivity. Men with university education reported less frequent pain than men with lower levels of education, possibly as they were more likely to have non-manual occupations. All kinds of unfavorable working positions may lead to frequent pain, as are occupations known to involve heavy manual work [12].

Smoking and AUD were positively associated with frequent pain, presumably as coping mechanisms for the latter [43, 44]. Previous studies have shown that alcohol is commonly used by construction workers [30], but the prevalence of AUD was low in this data set since we only included diagnoses from the hospital registry, which only covers the most severe cases. Both sleep problems and depressive symptoms were more prevalent among men reporting physical pain, which strengthens the internal validity of the pain question [45] and suggests a mechanism for how pain may increase the risk for suicidal behavior [46, 47].

### Prospective associations between pain and non-fatal self-harm/suicide

Construction workers with frequent pain in at least one location had twice the risk of suicide compared to workers without pain during the past year, which is consistent with findings from other populations [22, 24]. Risk of non-fatal self-harm among workers with frequent pain was 25% higher than among pain-free subjects; this finding is slightly weaker than the results of a recent meta-analysis, which, however, did not include prospective data and showed large heterogeneity between the studies [23].

Lower back pain was the most common location of pain but was not associated with suicidal behavior in this population. However, pain in the upper and lower extremities were independent risk factors for suicidal behavior. Although pain and functional impairment in hands and feet have been associated with poor mental health [48, 49] and risk of intentional self-harm [50] in patient groups, this is the first time that the risk of pain in different anatomical locations and suicidal behavior has been investigated in a large cohort of workers who were not selected on the basis of their pain experiences. Pain in the hands and feet is often a sign of chronic conditions such as arthritis, diabetes, or nerve damage caused by exposure to hand-arm vibrations [48, 51]. These conditions are difficult to treat and can lead to permanent impairment of mobility and dexterity. Back pain, on the other hand, may subside within a few months and may have less long-term impact on quality of life and mental health than pain in the extremities. While frequent pain in elbow, hand, or wrist increased the risk of suicide by 50%, we found no association between frequent leg pain and suicide death. It is likely that normal arm and hand function may be more salient to mental health, as suggested by previous research involving industrial workers who underwent amputation following workplace accidents. Upper limb amputation was more frequently associated with mental ill-health than lower limb amputation [52], the former leading to greater impairment in terms of self-care, expression, and communication than the latter [53]. The fact that the pain in the foot, ankle or knee was associated exclusively with non-fatal self-harm further suggests that the risk factors for fatal and non-fatal self-harm may differ [22, 54]. Consistent with our results, an earlier population-based study involving 17,148 Swedish twins found no increased risk of suicidal behavior associated with neck and shoulder pain [55], but further studies are needed to investigate the significance of pain location.

### Occupational factors and subsequent non-fatal self-harm/suicide

Despite considerable differences in the experience of pain across occupations, no occupational group showed a statistically significant increase in suicidal behavior after correction for multiple testing, although some groups (e.g., crane operators) had elevated risk estimates. Instead, a more general factor – lifting heavy loads - was associated with suicidal behavior; should this be confirmed in other studies, the assessment of poor posture at work could become a key component of health and safety measures for construction workers.

### Strengths and limitations

The long-term follow-up is a major strength of the study that allowed to investigate prospective associations between pain exposure and the relatively rare outcomes of suicide and non-fatal self-harm. As the exposure was assessed in the early nineties, prevalence estimates may not be considered relevant today. However, modern cohorts showed similar estimates [7, 10] and associations between pain and suicidal behavior are expected to be less affected by the absolute prevalence of the exposure. The lack of data on certain questions relating to pain does represent a limitation, but the imputation of ‘no exposure’ did not change conclusions about pain prevalence and pain-related associations. Although the lack of a validated measure for pain may be alleviated by observed associations with known correlates, access to primary care data for diagnoses of pain-related disorders would have strengthened the study. The risk estimates for frequent pain were generally higher for endpoints with determined intent, which strengthens the validity of the findings. Despite the high coverage of the Swedish inpatient register [56], the lack of information on non-fatal self-harm from primary care registers represents a limitation. Since risk factors were assessed cross-sectionally, we were unable to assess the extent to which the association between physical pain and suicidal behaviour was mediated by possible coping mechanisms such as smoking and alcohol consumption, or by sleep disturbances and depressive symptoms. To reduce the risk of reverse causation, all events occurring up to two years after baseline were excluded, but bidirectional associations between pain and mental health cannot be ruled out completely [17].

### Conclusions and need for future research

In this nationwide cohort of male construction workers followed for up to three decades, frequent physical pain was common and associated with an increased long-term risk of both non-fatal self-harm and suicide. These associations were most consistent for pain in the upper and lower limbs. The findings suggest that physical pain should be recognized as an indicator of vulnerability to suicidal behaviour, and that pain and mental health should be treated together rather than through separate care pathways [14, 57]. Consultations for physical pain in occupational health and primary care can provide an opportunity to also assess psychological distress and mental health. This may be particularly pertinent in the context of persistent limb pain, which may reflect chronic conditions with lasting functional impairment [48, 51]. Given the stigma surrounding help-seeking in the construction sector [30], such consultations may be one of few opportunities for early identification of mental ill-health. Brief questions about mood, sleep, alcohol use and suicidal thoughts could be considered, as previously proposed for podiatry outpatients [49]. At the workplace level, reducing exposure to heavy lifting, non-ergonomic postures and hand-arm vibration remains essential for preventing pain and may also benefit mental health. Given our observational design, these implications should be regarded as hypothesis-generating. In addition, longitudinal studies are needed to distinguish between transient and persistent pain and to provide data on key covariates over time, including use of analgesics, alcohol consumption, smoking, sleep problems, symptoms of depression and suicidal ideation and behavior. Further, studies in other contexts —including women, and different occupations and cultural settings—are needed to assess the generalisability of our results. Mechanisms underlying pain in the upper and lower limbs warrant direct investigation, ideally incorporating measures of functional impairment, work ability, and withdrawal from the labor market. Finally, intervention studies are required to determine whether strategies that effectively relieve pain also lead to lower rates of suicidal behaviour.

## Supplementary Information

Below is the link to the electronic supplementary material.

Supplementary Material 1

## Data Availability

No datasets were generated or analysed during the current study.

## Declarations

### Protection of research participants

Participants’ personal data were protected through anonymization and secure storage of data files. Furthermore, no information about individual participants or small subgroups that could lead to the identification of individuals was presented in the publication.
