# Supplementary material for: Physical pain and risk of suicide and non-fatal self-harm among Swedish construction workers followed over three decades
Source: J Occup Med Toxicol. 2026 Oct 12;21:34. doi: 10.1186/s12995-026-00532-6 (PMC13663277; doi:10.1186/s12995-026-00532-6)
Supplement: Supplementary file 1 [file 12995_2026_532_MOESM1_ESM.docx]

**SUPPLEMENT**

**Table S1**: Creation of the analytic sample for male construction workers with questionnaire data in 1989 – 1993.

| N | Exclusion criteria |
| --- | --- |
| 389,132 |  |
|  | Examined before 1989 (-234,550) |
| 154,582 |  |
|  | No questionnaire data (-67,477) |
| 87,105 |  |
|  | Female sex (- 3,459) |
| 83,646 |  |
|  | Age at completion of questionnaire > 64 years (-350) |
| 83,296 |  |
|  | Inconsistent response (yes to all questions, -7) |
| 83,289 |  |
|  | Missing data for pain frequency at all 9 locations (-104) |
| 82,678 |  |
|  | Events of non-fatal self-harm before baseline (- 415) |
| 82,263 |  |
|  | Events (any self-harm or censoring) within 2 years from baseline examination (- 3,164) |
| **79,099** |  |

**Table S2**: Frequency of pain at nine locations as well as the overall maximum in the overall sample and separately for subjects with and without missing data for pain.

|  | Frequency of pain over the past year, n (row %) | | | | |
| --- | --- | --- | --- | --- | --- |
| Location of pain | Never | Rarely | Sometimes | Often | Very often |
| Neck ^a^ | 31,927  (40) | 14,352  (18) | 20,013  (25) | 7,680  (10) | 5,127  (6) |
| Shoulder ^a^ | 29,972  (38) | 12,658  (16) | 20,132  (25) | 9,545  (12) | 6,792  (9) |
| Elbow ^a^ | 49,130  (62) | 11,728  (15) | 10,539  (13) | 4,322  (5) | 3,380  (4) |
| Wrist, hand ^a^ | 42,413  (54) | 14,044  (18) | 14,540  (18) | 4,946  (6) | 3,156  (4) |
| Upper back ^a^ | 42,012  (53) | 18,118  (23) | 13,170  (17) | 3,826  (5) | 1,973  (2) |
| Lower back ^a^ | 20,119  (25) | 11,607  (15) | 27,862  (35) | 12,090  (15) | 7,421  (9) |
| Hip ^a^ | 53,957  (68) | 11,661  (15) | 8,357  (11) | 3,059  (4) | 2,065  (3) |
| Knee ^a^ | 30,271  (38) | 11,850  (15) | 21,622  (27) | 9,266  (12) | 6,090  (8) |
| Ankle, foot ^a^ | 50,947  (64) | 13,064  (17) | 9,130  (12) | 3,403  (4) | 2,555  (3) |
| Maximal pain frequency  in at least one location ^a^ | 5,130  (6) | 4,083  (5) | 26,196  (33) | 21,970  (28) | 21,720  (27) |
| Maximal pain frequency in at least one location |  |  |  |  |  |
| - in the subset without missing data (n = 73,013) | 5,073  (7) | 3,995  (5) | 24,370  (33) | 19,993  (27) | 19,582  (27) |
| - in the subset with 1-8 unanswered questions   (n = 6,086) | 57  (1) | 88  (1) | 1,826  (30) | 1,977  (32) | 2,138  (35) |

^a^ data for Figure 1B

**Table S3:** Prevalence of frequent pain in at least one location and in specific locations by occupation

|  | Total | **In at least one location** | Neck | Shoulder | Elbow | Wrist, hand | Upper back | Lower back | Hip | Knee | Ankle, foot |
| --- | --- | --- | --- | --- | --- | --- | --- | --- | --- | --- | --- |
|  | N | **Row % ^a^** | | | | | | | | | |
| 1 Road builder | 1,160 | **60** | 19 | 26 | 12 | 11 | 7 | 28 | 9 | 16 | 7 |
| 2 Rock, ground worker | 2,774 | **54** | 12 | 18 | 9 | 10 | 7 | 27 | 8 | 18 | 7 |
| 3 Concrete worker | 5,555 | **60** | 16 | 23 | 12 | 13 | 8 | 29 | 9 | 20 | 9 |
| 4 Woodworker, roofer | 18,056 | **57** | 15 | 20 | 11 | 11 | 7 | 26 | 6 | 20 | 8 |
| 5 Bricklayer | 2,077 | **61** | 16 | 23 | 13 | 10 | 8 | 30 | 8 | 22 | 7 |
| 6 Floor-layer | 1,470 | **61** | 15 | 22 | 10 | 13 | 8 | 33 | 7 | 28 | 9 |
| 7 Machine operator | 2,179 | **55** | 22 | 27 | 10 | 10 | 8 | 26 | 9 | 14 | 6 |
| 8 Crane operator | 717 | **62** | 33 | 32 | 12 | 10 | 11 | 27 | 8 | 15 | 8 |
| 9 Driver | 681 | **58** | 19 | 24 | 13 | 12 | 9 | 28 | 9 | 16 | 8 |
| 10 Glass worker | 618 | **55** | 15 | 20 | 10 | 13 | 9 | 23 | 6 | 20 | 8 |
| 11 Insulation worker, plumber | 5,769 | **61** | 17 | 22 | 10 | 12 | 8 | 26 | 7 | 29 | 8 |
| 12 Reparation, refrigeration mechanic | 918 | **54** | 16 | 19 | 7 | 9 | 7 | 25 | 5 | 19 | 9 |
| 13 Painter | 5,745 | **58** | 24 | 26 | 10 | 10 | 9 | 25 | 5 | 20 | 7 |
| 14 Sheet-metal worker | 2,933 | **62** | 15 | 23 | 12 | 15 | 9 | 27 | 6 | 26 | 9 |
| 15 Electrician | 9,145 | **52** | 17 | 19 | 8 | 10 | 7 | 20 | 4 | 19 | 7 |
| 16 Foreman, clerk | 10,424 | **44** | 12 | 14 | 6 | 5 | 5 | 18 | 6 | 12 | 7 |
| 17 Other | 6,074 | **53** | 15 | 19 | 9 | 10 | 8 | 24 | 7 | 18 | 9 |
| Overall (ref) | 76,295 | **55** | 16 | 20 | 10 | 10 | 7 | 25 | 6 | 19 | 8 |

Multiple logistic regression of frequent pain on occupational group adjusted for year, region, and age at examination; results for individual occupational groups compared to the overall mean are color-coded with OR > 1 in red and OR < 1 in green. Only results with p-values <.003 are marked as statistically significant, to account for multiple testing.

^a^ The sum of row percentages for frequent pain in different locations is not bound by 100% as the locations are not mutually exclusive.

**Table S4:** Mean age as well as prevalence of frequent unfavorable working positions by occupation (row %, N = 78,750)

|  |  | Unfavorable working position | | | |
| --- | --- | --- | --- | --- | --- |
|  | Age | Heavy lifting | Bent, twisted | Hands above shoulders | Kneeling |
|  | Mean (SD) | Row (%) ^a^ | | | |
| 1 Road builder | 40 (11) | 57 | 71 | 21 | 18 |
| 2 Rock, ground worker | 38 (11) | 63 | 69 | 14 | 50 |
| 3 Concrete worker | 39 (12) | 66 | 67 | 44 | 59 |
| 4 Woodworker, roofer | 35 (11) | 58 | 56 | 58 | 64 |
| 5 Bricklayer | 38 (13) | 65 | 82 | 70 | 72 |
| 6 Floor-layer | 35 (11) | 78 | 81 | 50 | 97 |
| 7 Machine operator | 41 (11) | 14 | 20 | 6 | 7 |
| 8 Crane operator | 44 (10) | 18 | 38 | 13 | 9 |
| 9 Driver | 42 (11) | 40 | 33 | 20 | 13 |
| 10 Glass worker | 35 (11) | 70 | 65 | 47 | 43 |
| 11 Insulation worker, plumber | 36 (12) | 59 | 73 | 75 | 85 |
| 12 Reparation, refrigeration mechanic | 38 (11) | 51 | 58 | 41 | 62 |
| 13 Painter | 36 (12) | 39 | 62 | 90 | 79 |
| 14 Sheet-metal worker | 34 (11) | 56 | 75 | 61 | 79 |
| 15 Electrician | 34 (11) | 26 | 58 | 82 | 77 |
| 16 Foreman, clerk | 43 (10) | 7 | 10 | 7 | 10 |
| 17 Other | 36 (13) | 49 | 53 | 40 | 43 |
| Overall | 37 (12) | 45 | 54 | 51 | 57 |

Multiple linear regression of age at examination on occupational group adjusted for year and region as well as multiple logistic regression of frequent working position on occupational group adjusted for year, region, and age at examination; results for individual occupational groups compared to the overall mean are color-coded with OR > 1 in red and OR < 1 in green. Only results with p-values <0.003 are marked as statistically significant, to account for multiple testing.

^a^ The sum of row percentages for different working positions is not bound by 100% as the positions are not mutually exclusive.

**Table S5**: Pain and risk of suicidal behavior adjusted for different sets of potential confounders among subjects with complete data on the pain question (HR with 95% CI)

|  | Non-fatal  self-harm | Non-fatal self-harm with  known intent | Suicide | Suicide with known intent |
| --- | --- | --- | --- | --- |
| ***Model set 1: Adjustment for year, region, and age at baseline*** | | | | |
| Number of events /totals | 1,375 /73,013 | 409 /73,013 | 250 /73,013 | 218 /73,013 |
| ***1A.* *Maximum pain frequency in at least one location (ref = never)*** | | | | |
| Occasional | 1.03 (0.83, 1.28) | 1.14 (0.75, 1.72) | 1.70 (0.90, 3.22) | 1.82 (0.90, 3.70) |
| Frequent | **1.24 (1.00, 1.53)** | 1.40 (0.93, 2.10) | **1.96 (1.05, 3.69)** | **2.13 (1.06, 4.29)** |
| ***1B. Maximum pain frequency in specified regions (ref = never)*** | | | | |
| *Neck, shoulder, upper back* |  |  |  |  |
| Occasional | 0.97 (0.83, 1.12) | 1.07 (0.81, 1.38) | 1.43 (0.99, 2.06) | 1.40 (0.94, 2.07) |
| Frequent | 0.98 (0.83, 1.17) | 1.03 (0.75, 1.42) | 1.18 (0.77, 1.82) | 1.18 (0.75, 1.87) |
| *Elbow, wrist/hand* |  |  |  |  |
| Occasional | **1.19 (1.05, 1.35)** | 1.16 (0.92, 1.46) | 1.33 (0.98, 1.80) | 1.27 (0.92, 1.75) |
| Frequent | 1.15 (0.97, 1.36) | 1.27 (0.94, 1.72) | **1.64 (1.13, 2.39)** | **1.63 (1.09, 2.42)** |
| *Lower back, hip* |  |  |  |  |
| Occasional | 1.06 (0.92, 1.23) | 0.91 (0.70, 1.18) | 0.81 (0.58, 1.14) | 0.82 (0.57, 1.18) |
| Frequent | 1.13 (0.95, 1.34) | 0.97 (0.71, 1.33) | 0.96 (0.65, 1.43) | 0.92 (0.60, 1.40) |
| *Knee, ankle/foot* |  |  |  |  |
| Occasional | 1.05 (0.92, 1.20) | 1.13 (0.88, 1.46) | 1.04 (0.77, 1.42) | 1.13 (0.81, 1.58) |
| Frequent | **1.24 (1.06, 1.44)** | **1.58 (1.20, 2.09)** | 0.86 (0.59, 1.26) | 1.04 (0.70, 1.55) |
| ***Model set 2: Model set 1 with adjustment for parental origin, SBP, height, BMI, education, marital status*** | | | | |
| Number of events /totals | 1346 /70,571 | 405 /70,571 | 245 /70,571 | 214 /70,571 |
| ***2A.* *Maximum pain frequency in at least one location (ref = never)*** | | | | |
| Occasional | 1.01 (0.81, 1.26) | 1.11 (0.74, 1.68) | 1.68 (0.89, 3.19) | 1.81 (0.89, 3.68) |
| Frequent | 1.21 (0.97, 1.50) | 1.36 (0.91, 2.05) | **2.02 (1.07, 3.81)** | **2.18 (1.08, 4.40)** |
| ***2B. Maximum pain frequency in specified regions (ref = never)*** | | | | |
| Neck, shoulder, upper back |  |  |  |  |
| Occasional | 0.97 (0.84, 1.12) | 1.06 (0.81, 1.39) | 1.37 (0.95, 1.98) | 1.35 (0.91, 1.20) |
| Frequent | 0.97 (0.82, 1.16) | 1.04 (0.75, 1.43) | 1.14 (0.74, 1.76) | 1.14 (0.72, 1.81) |
| Elbow, wrist/hand |  |  |  |  |
| Occasional | **1.18 (1.04, 1.33)** | 1.13 (0.89, 1.42) | **1.35 (1.00, 1.83)** | 1.27 (0.92, 1.77) |
| Frequent | 1.13 (0.95, 1.34) | 1.26 (0.93, 1.70) | **1.68 (1.15, 2.46)** | **1.64 (1.09, 2.45)** |
| Lower back, hip |  |  |  |  |
| Occasional | 1.05 (0.90, 1.21) | 0.90 (0.69, 1.17) | 0.82 (0.58, 1.16) | 0.84 (0.58, 1.22) |
| Frequent | 1.10 (0.92, 1.31) | 0.94 (0.69, 1.29) | 1.00 (0.67, 1.49) | 0.96 (0.63, 1.48) |
| Knee, ankle/foot |  |  |  |  |
| Occasional | 1.06 (0.93, 1.21) | 1.14 (0.89, 1.47) | 1.06 (0.78, 1.45) | 1.13 (0.81, 1.59) |
| Frequent | **1.24 (1.06, 1.45)** | **1.58 (1.20, 2.10)** | 0.88 (0.60, 1.29) | 1.05 (0.70, 1.58) |
| ***Model set 3: Model set 2 with adjustment for occupational group and unfavorable working positions*** | | | | |
| Number of events /totals | 1328 /69,221 | 404 /69,221 | 240 /69,221 | 209 /69,221 |
| ***3A.* *Maximum pain frequency in at least one location (ref = never)*** | | | | |
| Occasional | 1.02 (0.82, 1.28) | 1.08 (0.71, 1.63) | 1.61 (0.85, 3.07) | 1.73 (0.85, 3.53) |
| Frequent | 1.20 (0.96, 1.50) | 1.24 (0.82, 1.88) | 1.88 (0.99, 3.57) | 2.01 (0.98, 4.10) |
| ***3B. Maximum pain frequency in specified regions (ref = never)*** | | | | |
| Neck, shoulder, upper back |  |  |  |  |
| Occasional | 0.97 (0.84, 1.13) | 1.06 (0.81, 1.40) | **1.49 (1.02, 2.18)** | 1.48 (0.99, 2.23) |
| Frequent | 0.97 (0.81, 1.16) | 1.02 (0.73, 1.41) | 1.28 (0.82, 2.00) | 1.29 (0.80, 2.08) |
| Elbow, wrist/hand |  |  |  |  |
| Occasional | **1.17 (1.03, 1.33)** | 1.11 (0.88, 1.40) | 1.31 (0.96, 1.79) | 1.23 (0.88, 1.72) |
| Frequent | 1.12 (0.94, 1.34) | 1.22 (0.90, 1.65) | **1.62 (1.10, 2.39)** | **1.58 (1.05, 2.38)** |
| Lower back, hip |  |  |  |  |
| Occasional | 1.03 (0.89, 1.20) | 0.87 (0.67, 1.14) | 0.78 (0.55, 1.11) | 0.81 (0.56, 1.18) |
| Frequent | 1.08 (0.90, 1.29) | 0.88 (0.64, 1.21) | 0.92 (0.62, 1.39) | 0.89 (0.58, 1.39) |
| Knee, ankle/foot |  |  |  |  |
| Occasional | 1.06 (0.93, 1.22) | 1.12 (0.87, 1.45) | 1.05 (0.76, 1.43) | 1.10 (0.78, 1.55) |
| Frequent | **1.24 (1.06, 1.45)** | **1.55 (1.17, 2.06)** | 0.86 (0.58, 1.27) | 1.01 (0.67, 1.53) |

Cox proportional hazard regression models adjusted for different sets of baseline covariates; statistically significant results are marked in bold (p <.05).
